# Impact of method and duration of post discharge surveillance on detection of surgical site infections

**DOI:** 10.1186/1753-6561-5-S6-O60

**Published:** 2011-06-29

**Authors:** M Koek, J Wille, A Voss, B van Benthem, M Isken

**Affiliations:** 1 CIb, RIVM, Bilthoven, Netherlands; 2 CBO, Utrecht, Netherlands; 3 Medical Microbiology & Infectious Diseases, Canisius-Wilhelmina Hospital, Netherlands; 4 Medical Microbiology & Infectious Diseases, Radboud University Nijmegen Medical Centre, Nijmegen, Netherlands

## Introduction / objectives

Since surgical site infections (SSIs) arise before and after discharge from the hospital, post discharge surveillance (PDS) of SSIs is inevitable for proper surveillance. Two methods of PDS are recommended by the Dutch surveillance network (PREZIES). Duration of PDS normally is 30 days or 1 year after surgery for implant-free and implant surgery respectively. We compare cumulative SSI rates over time for “recommended PDS” and “other PDS-methods”, and investigate whether the advised duration of PDS is justifiable.

## Methods

From PREZIES data (1999-2008) four implant-free surgical procedures (breast amputation, caesarean section, cholecystectomy and colon resection) and two implant surgeries (hip and knee replacement) were selected. Using survival techniques and Cox regression analyses SSI rates over time were studied and relative risks (hazard rates, HR) to detect SSIs were calculated for the PDS methods for several periods of time.

## Results

105,607 cases were collected from 87 hospitals. HRs to detect SSIs were significantly increased for recommended PDS for 5 out of 6 procedures. For implant-free procedures this was mainly caused by high HRs for superficial SSIs. For 2 out of 4 implant-free procedures at least 10% of all SSIs was detected in the final period (day 22-30) while for knee and hip replacement only 2.1% and 1.3% of all SSIs was detected in the final period (months 10-12).

## Conclusion

The use of recommended PDS leads to better detection of SSIs, especially of superficial SSIs in implant-free procedures. For implant-free surgeries a 30 day PDS seems justified. For surveillance of hip and knee replacement a PDS duration of 1 year seems less justifiable. It could be worth to consider shortening the PDS duration for these two surgical procedures.

## Disclosure of interest

None declared.

